# Screening and evaluation of metabolites binding PRAS40 from Erxian decoction used to treat spinal cord injury

**DOI:** 10.3389/fphar.2024.1339956

**Published:** 2024-01-22

**Authors:** Li Lin, Jingchuan Yan, Jin Sun, Jianfeng Zhang, Bo Liao

**Affiliations:** ^1^ Department of Orthopedics, Tangdu Hospital, Air Force Military Medical University, Xi’an, Shaanxi, China; ^2^ Department of Pharmacy, Eighth Hospital of Xi’an City, Xi’an, Shaanxi, China

**Keywords:** spianl cord injury, Erxian decotion, autophagy, m-TOR, PRAS40

## Abstract

**Objective:** The PRAS40 is an essential inhibitory subunit of the mTORC1 complex, which regulates autophagy. It has been suggested that Erxian Decoction (EXD) could treat spinal cord injury (SCI) via the autophagy pathway. However, the mechanism of whether EXD acts through PRAS40 remains unclear.

**Methods:** With the help of immobilized PRAS40, isothermal titration calorimetry (ITC) and molecular docking, the bioactive metabolites in the EXD were screened. To establish *in vitro* SCI models, PC12 cells were exposed to hydrogen peroxide (H2O2) and then treated with the identified EXD substances. Furthermore, Western blot assay was carried out to identify potential molecular mechanisms involved. For assessing the effect of metabolites *in vivo*, the SCI model rats were first pretreated with or without the metabolite and then subjected to the immunohistochemistry (IHC) staining, Basso, Beattie & Bresnahan (BBB) locomotor rating scale, and H&E staining.

**Results:** The immobilized PRAS40 isolated indole, 4-nitrophenol, terephthalic acid, palmatine, sinapinaldehyde, and 3-chloroaniline as the potential ligands binding to PRAS40. Furthermore, the association constants of palmatine and indole as 2.84 × 106 M-1 and 3.82 × 105 M-1 were elucidated via ITC due to the drug-like properties of these two metabolites. Molecular docking results also further demonstrated the mechanism of palmatine binding to PRAS40. Western blot analysis of PC12 cells demonstrated that palmatine inhibited the expression of p-mTOR by binding to PRAS40, activating the autophagic flux by markedly increasing LC3. The injection of palmatine (10μM and 20 μM) indicated notably increased BBB scores in the SCI rat model. Additionally, a dose-dependent increase in LC3 was observed by IHC staining.

**Conclusion:** This research proved that EXD comprises PRAS40 antagonists, and the identified metabolite, palmatine, could potentially treat SCI by activating the autophagic flux.

## 1 Introduction

Spinal cord injury (SCI) is manifested with various functional deficits because of spinal cord axonal and neuronal damage (Hellenbrand et al., 2021). The National SCI Statistical Center data indicated that the incidence of SCI is approximately 17,000 new cases each year (Author anonymous, 2016). Therefore, research on effective SCI treatment is necessary. Previous studies have reported that SCI pathogenesis comprises multiple cellular and biochemical processes, including free radical formation (Hou et al., 2021), posttraumatic inflammatory response (Chen et al., 2021), autophagy (Ko et al., 2020), vascular ischemia (Ahn et al., 2020), apoptosis (Wang et al., 2019), and genetically programmed cell death (Xu et al., 2021). Autophagy is a cellular self-defense mechanism that prevents cell damage, promotes cell survival in the presence of nutrient deficiencies, and responds to cytotoxic stimuli (Klionsky et al., 2021). Studies have shown that promoting autophagy can reduce neuronal apoptosis, inhibit neuroinflammation, and promote functional recovery of the SCI (Rong et al., 2019). Therefore, regulating autophagy in neural tissue is expected to become an important means for treating SCI.

Roth *et al.* were the first to discover a Proline-rich Akt substrate of 40 kD (PRAS40) in 2003 and characterized it as a substrate of protein kinase B (PKB/Akt) (Kovacina et al., 2003). The literature indicates that PRAS40 is associated with the mammalian target rapamycin (mTOR) signaling pathways. PRAS40 directly binds to the mTOR kinase domain, thereby regulating mTOR activity (Ma et al., 2020). Among these, the mTOR signaling pathway has been suggested to be crucially associated with SCI pathogenesis (Ma et al., 2020; Vargova et al., 2021). Currently, the literature primarily focuses on discovering mTOR ligands, and studies on other protein components like PRAS40 are lacking (Szwed et al., 2021).

EXD, a traditional Chinese medicine (TCM), was introduced by Zhang Bo-Na in the early 1950s. It is frequently used as a traditional Chinese herbal prescription for menopausal syndrome and comprises six herbs: *Curculigo orchioides Gaertn [Hypoxidaceae; Curculiginis rhizoma], Epimedium brevicornu Maxim [Berberidaceae; Epimedii folium], Phellodendron chinense C.K.Schneid. [Rutaceae; Phellodendri chinensis cortex], Anemarrhena asphodeloides Bunge [Asparagaceae; Anemarrhenae rhizoma], Angelica sinensis (Oliv.) Diels [Apiaceae; Angelicae sinensis radix], Morinda officinalis F.C.How [Rubiaceae; Morindae officinalis radix]* (Li et al., 2007). Furthermore, EXD has revealed significant efficacy against many clinical diseases and has also been proven effective for treating SCI. The literature suggests that EXD could inhibit apoptosis and has neuroprotective effects in rats (Li et al., 2022). The mTOR signaling pathway has been linked with these pharmacodynamic effects, suggesting the presence of the PRAS40 antagonists in EXD.

In this investigation, the PRAS40 antagonists were screened from EXD using the immobilized PRAS40. *In-vivo* and *in-vitro* elucidation of the PRAS40 antagonists have been indicated as promising drug candidates for SCI treatment. This research identified bioactive metabolites from EXD and provided an alternate mechanism of EXD for treating SCI.

## 2 Materials and methods

### 2.1 Materials and reagents

The His-tagged PRAS40 protein was purchased from Genscript Co., Ltd (Nanjing, China). The herbs were obtained from Tongrentang Co., Ltd (Beijing, China). For reference, Indole (No. I104724) and palmatine chloride (No. P274975) were acquired from Aladdin Co., Ltd (Shanghai, China). All chemicals were analytically pure unless stated otherwise. MHY1485 (MCE, Monmouth Junction, NJ, United States), an agonist of mTOR. KingFisher magnetic particle processor (Thermo Fisher Scientific) was utilized for affinity selections, and the bioactive metabolites were identified *via* the UPLC-ESI-MS/MS system (UPLC, ExionLC™ AD).

### 2.2 Preparation of EXD

Weigh the herbs and add distilled water. After soaking for 30 min, boil for 30 min and then filter to obtain EXD. The ratio is shown in Table 1.

**TABLE 1:** Pharmaceutical ingredient of Erxian decoction.

Scientific name of plant^a^	Herb pinyin name	Family name	Dosage/300 mL (g)
*Curculigo orchioides Gaertn*	XIAN MAO	*Hypoxidaceae R.Br*	10
*Epimedium brevicornu Maxim*	Yin Yang Huo	*Berberidaceae Juss*	30
*Phellodendron chinense C.K.Schneid*	HUANG BO	*Rutaceae Juss*	12
*Anemarrhena asphodeloides Bunge*	ZHI MU	*Asparagaceae Juss*	10
*Angelica sinensis (Oliv.) Diels*	DANG GUI	*Apiaceae Lindl*	10
*Morinda officinalis F.C.How*	BA JI TIAN	*Rubiaceae Juss*	10

^a^ According to the Kew Herbarium Catalogue http://www.plantsoftheworldonline.org.

### 2.3 Affinity selection of bioactive metabolites from EXD

Briefly, the PRAS40 protein was incubated with Ni-NTA magnetic beads (10 mg) in PBS (pH 7.4) to coat the proteins for 30 min. Then, the beads were rinsed thrice with PBS + 1 mL Tween-20 (0.05% v/v, incubated for 1 h with EXD in PBS + Tween with mixing, and rinsed again with buffer + Tween 5 times to remove unbound metabolites. The resulting beads carrying bound metabolites were redissolved in elution buffer (10 mM imidazole, pH 8.5) to obtain the bioactive metabolites from EXD. These metabolites were analyzed using the Agilent column SB-C18 (2.1 mm × 100 mm, 1.8 µm) with the mobile phase comprising solvents A (0.1% formic acid + pure water) and B (0.1% formic acid + acetonitrile). For the identification of the bioactive metabolites, a linear gradient program from 95% A, 5% B to 5% A, 95% B within 9 min until a composition of 5% A, 95% B was kept for 1 min. The column was run at the temperature of 40°C, 2 μL injection volume, and the 0.35 mL/min flow rate. The effluent was alternatively connected to an ESI-triple quadrupole-linear ion trap (QTRAP)-MS.

### 2.4 High performance liquid chromatography (HPLC) determination of metabolites concentration

Thermo Scientific UltiMate 3,000 for HPLC quantification. Chromatographic conditions: palmatine and terephthalic acid: mobile phase:0.01 mol/L Potassium dihydrogen phosphate and methanol(1:1), flow rate:1 mL/min, detection wavelength was 270 nm, Sample Volume 10 μL. 4-Nitrophenol: mobile phase: water and methanol (0.35:0.65), flow rate:1 mL/min, detection wavelength was 280 nm, Sample Volume 10 μL. Sinapinaldehyde, Indole and 3-Chloroaniline: water and methanol (0.3:0.7), flow rate: 0.8 mL/min, detection wavelength was 270 nm, Sample Volume 10 μL. The contents of each component were calculated according to the standard curve and the peak area of the sample.

### 2.5 Isothermal titration calorimetry (ITC)

Before the experiment, each sample was placed in a vacuum machine for 10 min to remove gas from the sample. Use the sampling needle that comes with the instrument to draw 300 uL of PRAS40 (5 μM) into the sample pool, and use the titration needle to draw 60 uL of the metabolites (50 μM) as the titrant. The conditions were set as 20 repeated injections at 25°C with an interval of 165 s between each injection. The equilibrium dissociation constant (Kd), stoichiometry (n), enthalpy (ΔH) and entropy (ΔS) were obtained by nanoAnalyzer software. For subsequent analysis, the metabolite with the highest thermodynamic parameter was selected.

### 2.6 Molecular docking validation

The sdf files of ligand small molecules were found in the PubChem database and converted into a PDB file through OpenBabel3.1.1. For receptor structure preparation, the 3D structure of the PRAS40 was downloaded from the UniProt database (Uniprot: Q96B36) and subjected to hydrogenation, water removal, and charge addition through AutoDock Tools 1.5.6. And convert the format of active ingredient and target protein into pdbqt format. Finally, molecular docking was performed through AutoDock Vina 1.2.2.

### 2.7 Cell culture

PC12 cells were provided by Stem Cell Bank, Chinese Academy of Sciences (Shanghai, China). Cells were cultured in Rosewell Park Memorial Institute (RPMI)-1640 medium (Invitrogen, Carlsbad, CA, United States) containing 10% (v/v) fetal bovine serum (FBS, Gibco) and 1% (v/v) penicillin-streptomycin-glutamine in 100 cm^2^ cell culture flask. The flask was placed in a humidity incubator at 37°C with 5% CO2.

### 2.8 Cell viability assay

Viabilities of PC12 cells were measured using cell counting kit-8 (CCK-8) assay (Beyotime Biotechnology, Shanghai, China) in this research. Briefly, PC12 cells were propagated in 96-well plates and incubated in a humidified incubator at 37°C for 24 h. Then different concentrations of H_2_O_2_ added into plates to stimulate PC12 cells for 24 h. After that, PC12 cells were treated with CCK-8 (10 μL) solution in fresh RPMI 1640 for 25 min. Each well’s absorbance was assessed *via* a microplate reader (Infinite M200 PRO; Tecan) at 450 nm.

### 2.9 Western blot

Total proteins in PC12 cells were isolated using RIPA Lysis Buffer (Beyotime Biotechnology). The protein concentration was determined using a BCA Protein Assay Kit (Beyotime Biotechnology, Shanghai, China) with a full-wavelength functional microplate reader (Infinite M200Pro, Tecan, Switzerland). Then equal concentration Proteins were separated using 12.5% SDS–PAGE and transferred to nitrocellulose membranes. After blocking in 10% nonfat dry milk for 1 h, phosphorylated proteins were blocked using bovine serum albumin (5% BSA, room temperature) for 2 h. The membranes were incubated overnight at 4°C with the following primary antibodies: anti-phospho-mTOR-(Ser2448) (ZRB1553), anti-m-TOR(SAB5700687), anti-LC3 (SAB5701328) (Sigma Aldrich, United States), anti-phospho-PRAS40-(Thr246) (Cell Signaling) and anti-PRAS40(Cell Signaling)antibodies. The membrane was then incubated with the corresponding secondary antibody for 1 h. After three washes with PBST, the membrane was visualized using an ECL Western blot detection kit (Merck, United States). The average optical density of the images was analyzed using ImageJ.

### 2.10 Animals and surgeries

Adult Wistar rats (weight = 250 g, female) were acquired from the SPF Biotechnology Co., Ltd. [license number: SCXK (Jing) 2019-0010] and housed in ventilated cages (2-4/cage) at an environmentally controlled (22°C–24°C) conditions, a 12-h dark cycle, and *ad libitum* chow and water. The Care and Use of Laboratory Animals and ARRIVE guidelines were employed in a licensed laboratory for all animal investigations (license number: SYXK (Shaan) 2020-007). This investigation was authorized (No. IACUC-20211003) by the Welfare and Ethics Committee of the Laboratory Animal Center of Air Force Military Medical University, China. Rats were randomly divided into 5 groups(n = 8): SCI group; SCI+5 μM palmatine group; SCI+10 μM palmatine group; SCI+20 μM palmatine group; sham group.

Surgical treatment was performed as described previously (Li et al., 2022). Briefly, After anesthesia with 25 mg/kg sodium pentobarbital solution, laminectomy from T8 to T10 was performed through a midline incision in the thoracic spine. A 10 g impactor fell vertically on the surface of the exposed T9 spinal cord from a height of 50 mm, causing a contusion injury. Rats with a tail swing action were included in the experiment, otherwise excluded. In the sham operation group, only the T9 lamina was removed without vertical impact. The palmatine group was injected with palmatine 10 μL (5, 10, and 20 μM concentrations) in the intrathecal space after impact. The SCI group was also injected with an equal volume of saline. After surgery, assisted urination (twice a day) will be given until spontaneous urination is restored. The BBB scores were used to assess the locomotor ability of rats at the time of pre-operation and awakening(0), 1, 3, and 7 days.

### 2.11 Perfusion and immunofluorescence

The animals were perfused 7 days after SCI. 100 mg/kg of pentobarbital sodium solution was utilized for anesthetizing the rats; then, their spinal cord tissues were sampled, preserved in paraformaldehyde (4%), embedded in wax, sliced, deparaffinized, hydrated, and treated with sodium citrate antigen retrieval solution (1:50, Proteintech, United States). The samples were then blocked for 1 h using 5% sheep serum albumin (Boster Biological, China) in PBS at room temperature before overnight immunostaining with primary anti-LC3 antibodies and secondary antibodies. After mounting (DAPI Fluoromount-G, Southern Biotech, United States), the specimens were imaged via a confocal laser scanning microscope (Olympus BX51) and DP Controller software (Olympus, Japan).

### 2.12 Statistical analysis

The normality and homogeneity of variances were tested using SPSS (version 25.0). One-way analysis of variance (ANOVA) was utilized to analyze data from multiple groups. To determine differences between groups, multiple comparisons were conducted using Bonferroni *post hoc* tests. The error bars in all figures indicate the mean ± standard error of the mean (SEM). A significance level of *p* < 0.05 was considered statistically significant. Data analysis was performed using SPSS and GraphPad Prism (version 6.0c).

## 3 Results and discussion

### 3.1 Screening PRAS40 binding metabolites from EXD

**FIGURE 1:**
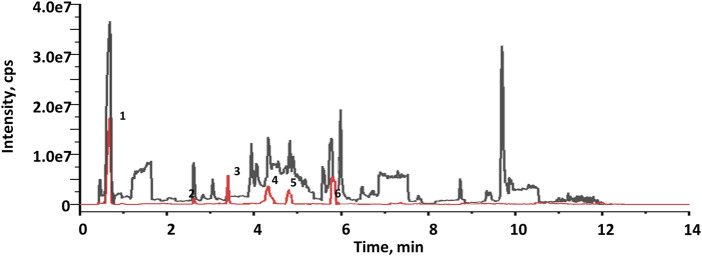
Total current ion chromatograms of EXD (black) and collections after screening using immobilized PRAS40 (red).

The EXD has been widely used for SCI treatment. Considering the pathological mechanism of mTOR, it was hypothesized that EXD has certain metabolites that interact with PRAS40. Therefore, PRAS40 was immobilized onto the surface of beads for screening the bioactive metabolites from EXD. As illustrated in Figure 1, six metabolites were identified according to the increased relative concentration in the samples (Table 2). Among these, 4-nitrophenol and 3-chloroaniline indicated poor drug-like properties due to a lack of compatibility with Lipinski’s rules (Chen et al., 2020). Whereas sinapinaldehyde and terephthalic acid were Pan-Assay Interference Compounds (PAINS), which could indicate positive readouts in biochemical assays *via* different mechanisms; however, such readouts are not linked with optimizable and processible compounds (Grigalunas et al., 2020). Therefore, palmatine and indole were selected to determine their association constants binding to PRAS40 using the ITC, a robust method for analyzing protein-ligand interactions.

**TABLE 2:** The mass spectral data of the bioactive PRAS40 binding metabolites using HPLC-MSESI-QTOF and MS/MS in positive mode.

Peak/metabolites	Proposed metabolites	RT(min)	Ionization mode	m/z	Molecular formula	Concentration(%)
1	4-Nitrophenol	0.68	[M + H]^+^	140.03	C_6_H_5_NO_3_	0.05–0.06
2	Indole	2.61	[M + H]^+^	117.06	C_8_H_7_N	0.01–0.02
3	Terephthalic acid	3.34	[M + H]^+^	168.03	C_8_H_6_O_4_	0.04–0.05
4	Palmatine	4.32	[M]^+^	352.15	C_21_H_22_NO_4_	0.08–0.09
5	Sinapinaldehyde	4.83	[M + H]^+^	208.07	C_11_H_12_O_4_	0.01–0.02
6	3-Chloroaniline	5.79	[M + H]^+^	128.03	C_6_H_6_ClN	0.05–0.06

### 3.2 Thermodynamic parameters and molecular docking of palmatine and indole binding to PRAS40

**FIGURE 2:**
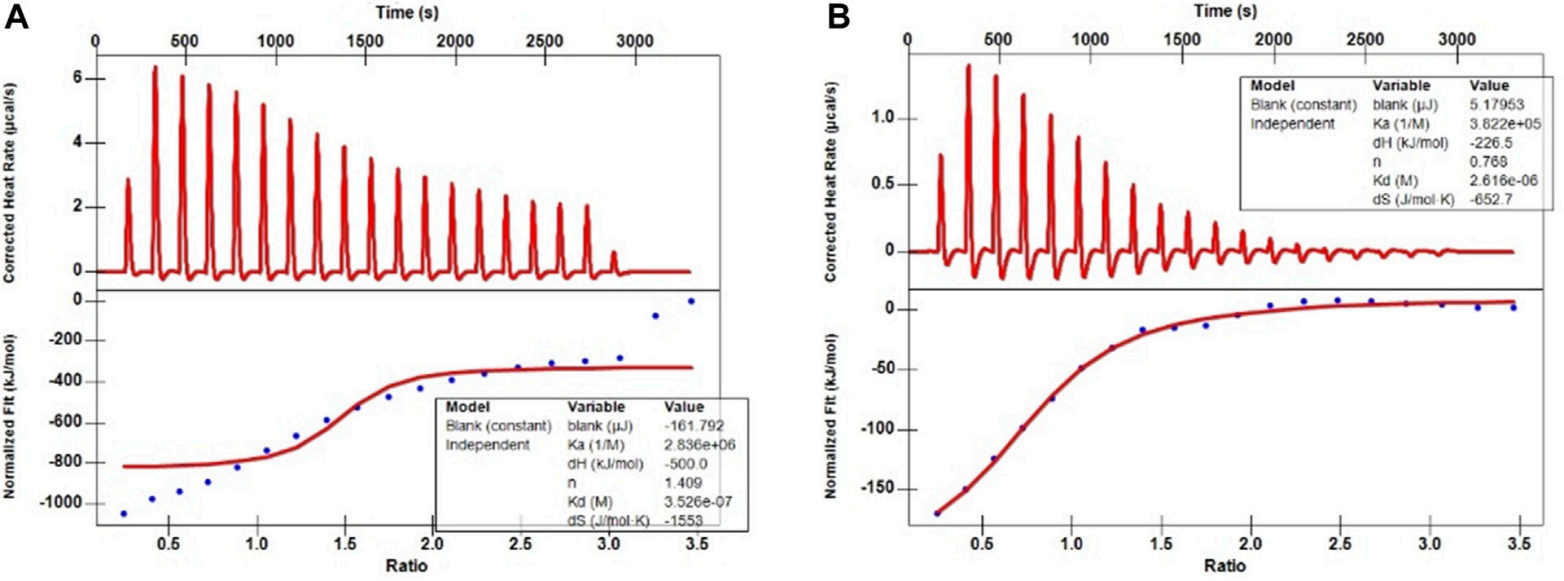
ITC traces and binding isotherms between the ligands and PRAS40. **(A)** Palmatine and PRAS40. **(B)** Indole and PRAS40.

The ITC aims to elucidate the heats of binding that evolve from protein-ligand interactions; however, in principle, titration yields other heats, too (Greytak et al., 2022). Therefore, first, the background heat between the solvent used to dissolve these two metabolites (5% DMSO in H_2_O) and the PBS buffer during titration was assessed. After deducting these background heats, the ITC traces and binding isotherms between the ligands and PRAS40 were obtained (Figure 2). The continuous line in the lower plots depicts a good data fit at a single-binding-site model used to acquire fitted parameters ( ∆G,∆H,and∆S ) values (Table 3). The resulting K_d_ values of palmatine and indole binding to PRAS40 could be calculated from the ∆G values. The K_d_ values of palmatine and indole were 3.53 × 10^−7^ and 2.62 × 10^−6^, respectively, indicating palmatine was a promising PRAS40 binding drug candidate for SCI treatment (Table 2). The higher K_a_ values of palmatine also confirmed the above conclusion.

**TABLE 3:** The ITC data of palmatine and indole binding with PRAS40.

Metabolites	K_a_ (M^-1^)	K_d_ (M)	∆H (KJ/mol)	∆S (J/mol·K)	∆G (KJ/mol)
Palmatine	2.84 × 10^6^	3.53 × 10^−7^	−500	−1553	−36.43
Indole	3.82 × 10^5^	2.62 × 10^−6^	−226.5	−625.7	−31.67

**FIGURE 3:**
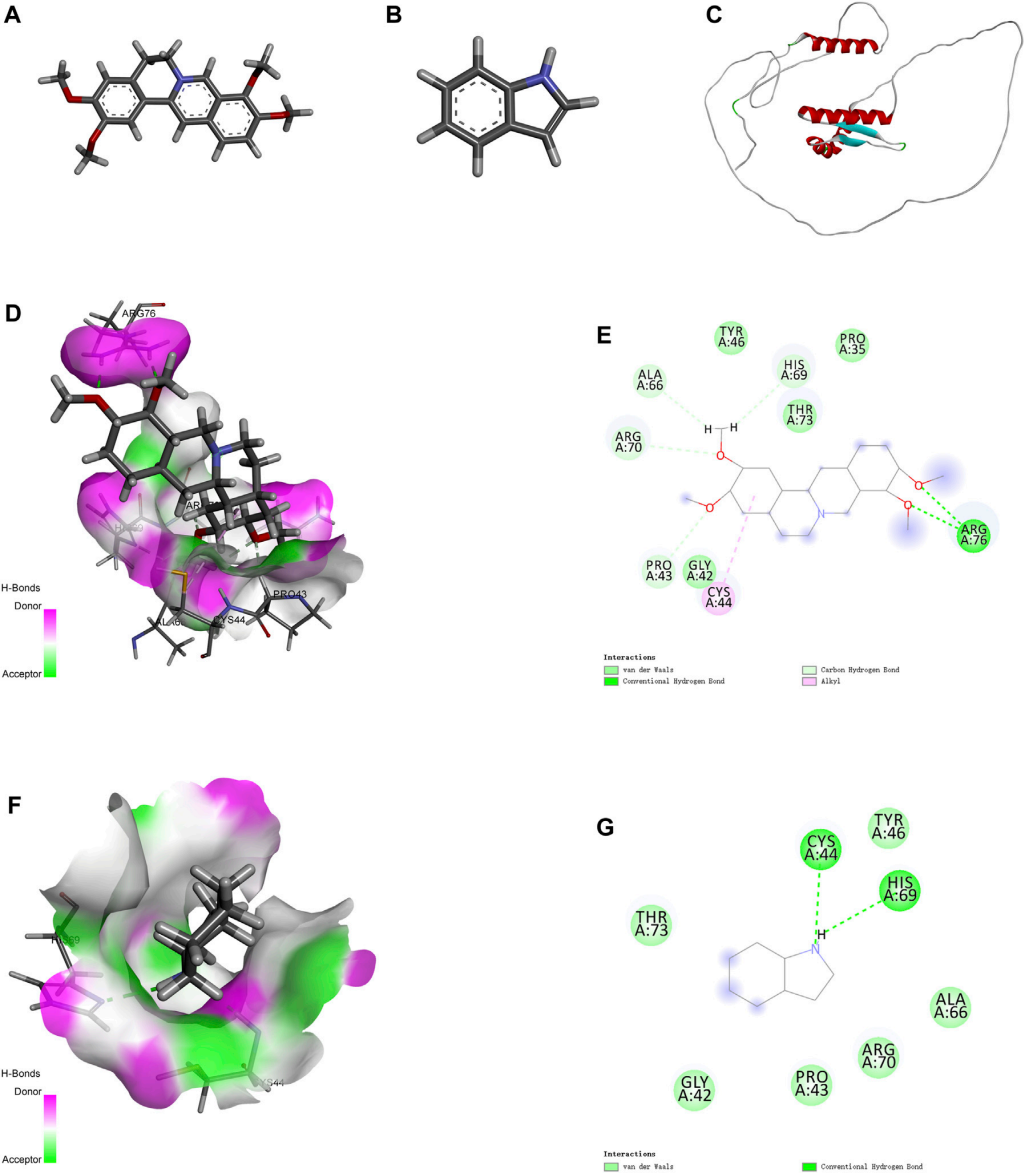
Molecular docking. **(A)** 2D structure of palmatine. **(B)** 2D structure of indole. **(C)** 3D structure of PRAS40. **(D)** Docking of PRAS40 to palmatine in 3D view, and **(E)** 2D view. **(F)** Docking of PRAS40 to indole in 3D view, and **(G)** 2D view.

Molecular docking is an effective approach for studying molecular interactions (Pinzi and Rastelli, 2019). Based on the above screening results, the 3D structure of PRAS40 was imported into AutoDock Tools and docked with palmatine and indole (Figures 3A–C). Molecular docking results showed that the binding energy of the PRAS40 to palmatine and indole was −5.06, −4.24 kcal/mol, indicating that the combination of palmatine and the protein is more stable. Among them, ARG76 of PRAS40 interacts with palmatine to form a conventional hydrogen bond interaction; PRO43, ARG70, ALA66, and HIS69 interact with palmatine to form a carbon-hydrogen bond interaction; CYS44 forms a Pi-Alkyl interaction with palmatine while GLY42, TYR46, THR43 and PRO35 generate van der Waals interactions with palmatine (Figures 3D–E). VAL190 of PRAS40 has a hydrogen bond interaction with indole, and CYS44 and HIS69 have a conventional hydrogen bond interaction. In addition, ALA66, ARG70, PRO43, GLY42, and THR73 of the protein have van der Waals interactions with indole (Figures 3F–G). The results of molecular docking are consistent with ITC. Therefore, the pharmacological activity of palmatine *in vitro* and *in vivo* was assessed.

### 3.3 Palmatine inhibits the phosphorylation of PRAS40 and activates autophagic flux in SCI cells model

**FIGURE 4:**
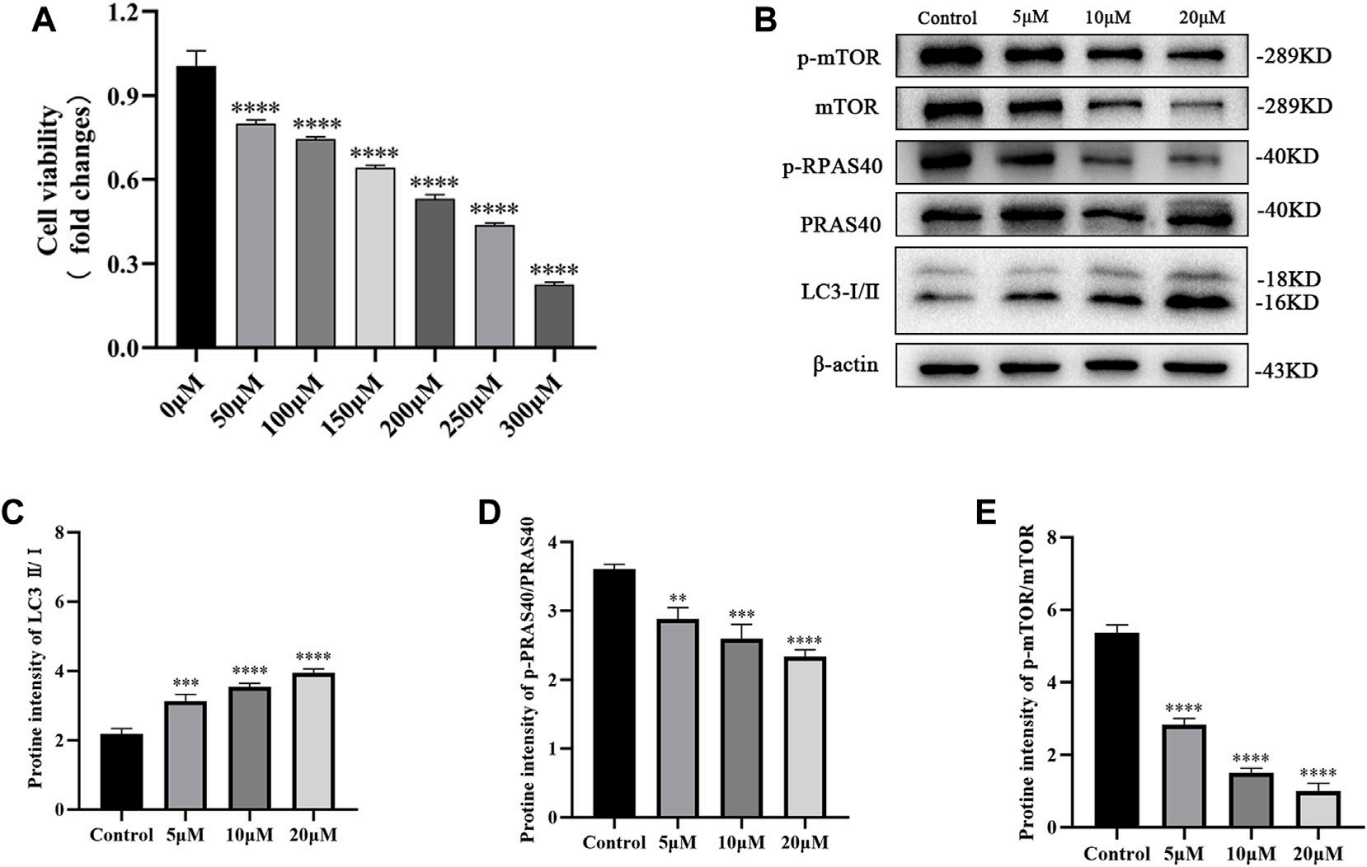
**(A)** PC12 cells viability after 24 h treatment with different concentrations of H_2_O_2_. **(B)** Western blot analysis for determining the expression of p-mTOR, mTOR, p-PRAS40, PRAS40, and LC3 (n = 3) **(C)** Quantification of the ratio of LC3-Ⅱ to LC3-Ⅰ(∗∗∗*p* < 0.001, ∗∗∗∗*p* < 0.0001), **(D)** p-PRAS40 to PRAS40 (∗∗*p* < 0.01, ∗∗∗*p* < 0.001, ∗∗∗∗*p* < 0.0001), and **(E)** p-mTOR to mTOR(∗∗∗*p* < 0.001, ∗∗∗∗*p* < 0.0001). The data are depicted as the mean ± SEM.

PC12 cells were stimulated with different H_2_O_2_ concentrations for 24 h to mimic SCI cells, which indicated a dose-dependent (0–300 μM) reduction in cell viability (Figure 4A). Based on this, 200 μM of H_2_O_2_ was selected for SCI construction (viability = 50%). Subsequently, the cells were treated with palmatine (5, 10, and 20 μM concentrations) for 2 h, which revealed a dose-dependent alleviation of the expression level of p-PRAS40, while the level of LC3 was increased (Figures 4B–E). At the same time, the phosphorylation of mTOR, an important target of PRAS40, is also downregulated as the drug concentration increases. Altogether, these data demonstrated that palmatine inhibited the expression of p-mTOR by binding to PRAS40, activating the autophagic flux of PC12 cells.

### 3.4 Palmatine can effectively repair motor function after SCI in rats

**FIGURE 5:**
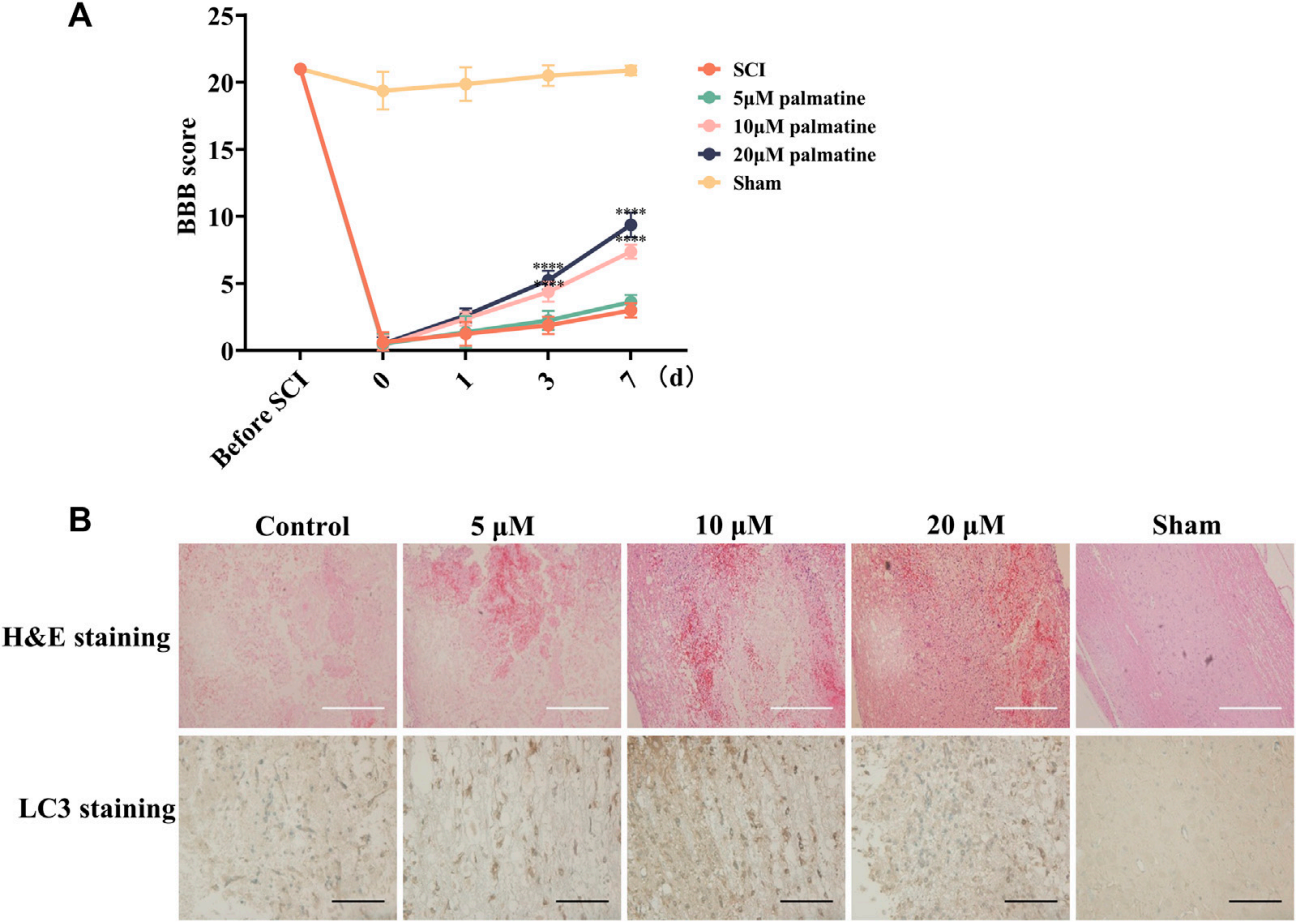
**(A)** Rats’s BBB scores at different time points (n = 8) ∗∗∗∗*p* < 0.0001. **(B)** H&E staining and Immunohistochemistry (IHC) staining using antibodies against LC3 in different groups (n = 3). White scale bar: 500 μm. Black scale bar: 100 μm.

Furthermore, the *in vivo* assessment was carried out by categorizing rats into five groups (n = 8/group). Following SCI, the treatment groups were injected with palmatine (5, 10, and 20 μM concentrations) in the intrathecal space. The remaining two groups were the sham-operated and negative control groups injected with saline. For each group, BBB scoring was performed during the 7 days treatment period. This score is negatively correlated with the severity of SCI. As illustrated in Figure 5A, palmatine at the two higher doses substantially increased the BBB scores than the control group, indicating a significant improvement in SCI rats. Lastly, the tumor tissue was harvested, and LC3 was quantitated by immunohistochemical staining. The treatment group indicated significantly positive expression of LC3, which was dose-dependently increased after palmatine treatment (Figure 5B). These results indicated the palmatine screened from EXD acts by activating the autophagic flux and could be a potential alternative mechanism explaining EXD used for SCI treatment.

### 3.5 Palmatine activates autophagic flux in SCI cells model by PRAS40/mTOR pathway

**FIGURE 6:**
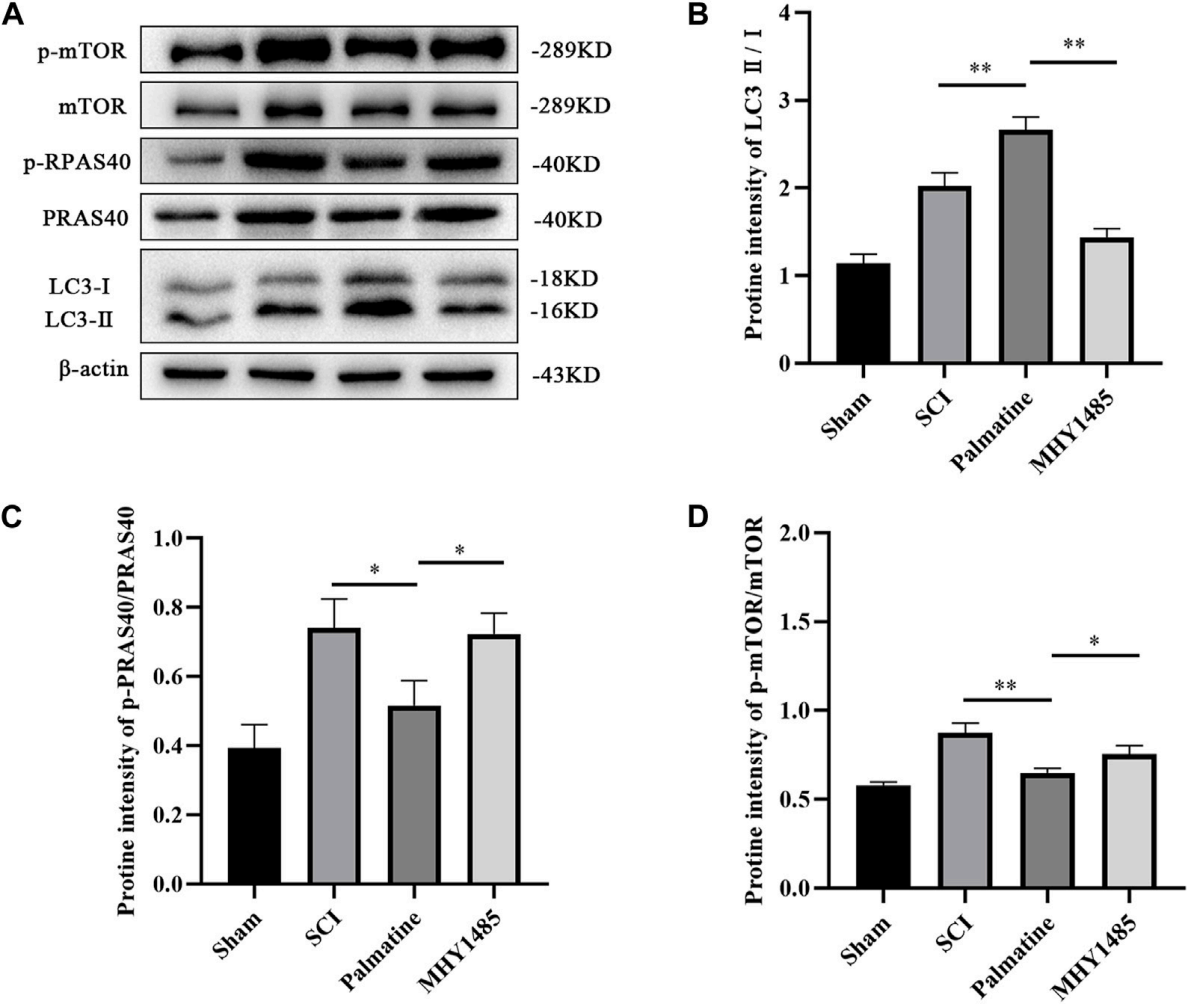
Palmatine activates autophagic flux by PRAS40/mTOR pathway. **(A)** Western blot analysis for determining the expression of p-mTOR, mTOR, p-PRAS40, PRAS40, and LC3 (n = 3) **(B)** Quantification of the ratio of LC3-Ⅱ to LC3-Ⅰ(∗∗*p* < 0.01), **(C)** p-PRAS40 to PRAS40 (∗*p* < 0.01), and **(D)** p-mTOR to mTOR(∗*p* < 0.01, ∗∗∗∗*p* < 0.001). The data are depicted as the mean ± SEM.

To further verify whether palmatine regulates autophagy through the PRAS40/mTOR pathway, PC12 cells were co-incubated with MHY1485 (an agonist of mTOR) and palmatine after treating with H_2_O_2_. The sham group was PC12 cells treated with saline. The results showed that palmatine treatment significantly promoted autophagy in SCI cells (Figure 6B). However, activation of mTOR by MHY1485 attenuated the effect of palmatine. Phosphorylation of PRAS40 can be inhibited by palmatine, as well as decreased p-mTOR levels and increased LC3 levels in the palmatine group compared to the H2O2 group (Figures 6C, D). However, when mTOR is activated, the expression of LC3, which represents the level of autophagy, decreases, indicating that the promotion effect of palmatine on autophagy was reversed.

## 4 Discussion

This investigation aimed to screen PRAS40 binding metabolites from EXD and evaluate the metabolite’s efficacy on functional repair after SCI. We first screened out the metabolites in EXD that are affinity with PRAS40 through ligand fishing and analyzed the kinetic parameters and mechanism of their binding to PRAS40 through ITC and molecular docking methods. Finally, we used BBB score, IHC, and WB to verify the effectiveness of the metabolites in treating SCI and it may regulate autophagy through the PRAS40/m-TOR pathway.

SCI refers to damage that causes temporary or permanent functional changes to the spinal cord. It is clinically characterized by high incidence, high cost, and high disability rates (Hu et al., 2023). Over the past few decades, researchers have adopted various strategies to reduce nerve damage and restore nerve function. Due to its long history and rich catalog of medical resources, TCM is also considered to be an effective means of clinical treatment for SCI (Lu et al., 2020). Therefore, studying the impact of TCM and its active extracts on SCI will help advance the treatment of SCI and the development of new clinical drugs. EXD, a famous TCM for the menopausal syndrome, was first introduced by Zhang Bo-Na in the early 1950s (Li et al., 2007). Our previous research showed that EXD can reduce spinal cord edema and improve motor function in SCI rats, suggesting EXD has the potential to treat SCI. In this study, we used the metabolites screened in EXD to treat SCI and improve the motor function of rats with SCI. This further provides support for EXD to be used in the clinical treatment of SCI.

Ligand fishing strategy is a complex system screening strategy based on affinity chromatography theory and using target immobilization technology (Zhuo et al., 2016). Ligand fishing is widely used to screen active compounds in complex samples due to its advantages of rapid and convenient screening (de Moraes et al., 2019; Chen et al., 2022). Finding drug leads from natural products will help subsequent clinical treatments. This study screened PRAS40 inhibitors in EXD by immobilizing PRAS40 and identified six metabolites according to the increased relative concentration (Figure 1; Table 2). By exclusion and screening, palmatine and indole were selected. Their association was determined using ITC and molecular docking. Based on the association constants of palmatine and indole as 2.84 × 106M-1 and 3.82 × 105 M-1, respectively, palmatine is a promising anti-PRAS40 drug candidate for treating SCI (Figure 2; Table 3). And subsequent molecular docking also confirmed these results. Molecular docking results showed that the binding energy of PRAS40 to palmatine and indole was −5.06 and −4.24 kcal/mol, and palmatine interacts with more amino acid residues of PRAS40 in a more abundant binding interaction (Figure 3).

Palmatine, a naturally occurring isoquinoline alkaloid, is a yellow compound found in various Chinese medicines, including *Berberidaceae, Papaveraceae, Ranunculaceae, and Menispermaceae* (Tarabasz and Kukula-Koch, 2020). In Chinese pharmacopeia, palmatine is considered an antibacterial and anti-inflammatory drug mainly used clinically for gynecological inflammation, enteritis, respiratory and urinary tract infections, surgical infections, etc (Johnson-Ajinwo et al., 2019; Ekeuku et al., 2020; Liu et al., 2021). The effects of palmatine on the nervous system have received widespread attention in recent years. Palmatine could pass the blood-brain barrier, improve cognitive impairment in 5xFAD mice after intraperitoneal injection, and alleviate the cerebellum and hippocampus proteome (Kiris et al., 2023). The administration of palmatine could effectively decrease the levels of pro-inflammatory cytokines and increase the levels of anti-inflammatory cytokines in LPS-induced BV2 cells (Wang et al., 2022). This investigation showed that in the SCI cell model palmatine can inhibit the phosphorylation of PRAS40 and increase the expression of LC3, a representative protein of autophagy, and this process is drug concentration-dependent(Figures 4A–D). Furthermore, palmatine significantly improved the BBB scores of SCI rats (Figure 5A). IHC staining of the SCI rat model also indicated that palmatine could treat SCI by regulating autophagy flux (Figure 5B).

Autophagy is an important pathway to maintaining internal balance in organisms, and the inhibition of mTOR physiologically controls the initiation of the autophagy cascade (Klionsky et al., 2021). Currently, there are inhibitors targeting mTOR that regulate the autophagy process. However, these drugs’ binding mode and mechanism of action to mTOR still need further study (Chen and Zhou, 2020). Furthermore, research has mainly focused on discovering mTOR ligands, while studies on other protein components that regulate the mTOR kinase domain are lacking. PRAS40 is an insulin-induced PKB/Akt substrate, also called “Akt1s1”. Its phosphorylated protein was first observed in the HeLa cell’s nuclear extracts (Beausoleil et al., 2004). PRAS40 is located on human chromosome 19q13.33, and its sequence is highly conserved among humans and other mammals (Macias et al., 2002; Wiza et al., 2012). Previous studies have shown that PRAS40 is a crucial inhibitory subunit of mTOR. It can suppress the mTOR kinase activity by binding to Raptor, a component of mTOR, thereby upregulating autophagy (Yamamura et al., 2022). The phosphorylation of PRAS40 leads to the separation of PRAS40 from mTORC1, which reduces the inhibition of mTORC1 (Zhou et al., 2021). In this study, we found that palmatine can regulate the expression of autophagy in SCI cell models. The activation of mTOR through MHY1485, which is an agonist of mTOR, proves that this process is achieved through the PRAS40/mTOR pathway (Figures 6A–D).

## 5 Limitations

However, there are several limitations. First, it was not determined whether other pathways are involved in the effects of palmatine on SCI. Second, The effectiveness and safety of palmatine in SCI treatment require further animal and clinical research support. Finally, The complexity of the EXD composition should be acknowledged, as different metabolites may have synergistic or antagonistic effects.

## 6 Conclusion

In summary, our study screened PRAS40 inhibitor palmatine from EXD. The *in vivo* and *in vitro* assays indicated that palmatine may treat SCI by regulating autophagy. We identified a novel mechanism that palmatine activates autophagy through the PRAS40/mTOR pathway, which also suggests that palmatine may be a new targeting mTOR inhibitor.

## Data Availability

The raw data supporting the conclusion of this article will be made available by the authors, without undue reservation.
